# Drug exposure during pregnancy: Current understanding and approaches to measure maternal-fetal drug exposure

**DOI:** 10.3389/fphar.2023.1111601

**Published:** 2023-03-23

**Authors:** Rachel E. Hudson, Torri D. Metz, Robert M. Ward, Autumn M. McKnite, Elena Y. Enioutina, Catherine M. Sherwin, Kevin M. Watt, Kathleen M. Job

**Affiliations:** ^1^ Division of Clinical Pharmacology, Department of Pediatrics, School of Medicine, The University of Utah, Salt Lake City, UT, United States; ^2^ Division of Maternal Fetal Medicine, Department of Obstetrics and Gynecology, School of Medicine, The University of Utah, Salt Lake City, UT, United States; ^3^ Department of Pharmacology and Toxicology, College of Pharmacy, The University of Utah, Salt Lake City, UT, United States; ^4^ Department of Pediatrics, Boonshoft School of Medicine, Wright State University, Dayton, OH, United States

**Keywords:** maternal-fetal pharmacology, pregnancy, fetal drug exposure, prenatal testing, pharmacokinetics

## Abstract

Prescription drug use is prevalent during pregnancy, yet there is limited knowledge about maternal-fetal safety and efficacy of this drug use because pregnant individuals have historically been excluded from clinical trials. Underrepresentation has resulted in a lack of data available to estimate or predict fetal drug exposure. Approaches to study fetal drug pharmacology are limited and must be evaluated for feasibility and accuracy. Anatomic and physiological changes throughout pregnancy fluctuate based on gestational age and can affect drug pharmacokinetics (PK) for both mother and fetus. Drug concentrations have been studied throughout different stages of gestation and at or following delivery in tissue and fluid biospecimens. Sampling amniotic fluid, umbilical cord blood, placental tissue, meconium, umbilical cord tissue, and neonatal hair present surrogate options to quantify and characterize fetal drug exposure. These sampling methods can be applied to all therapeutics including small molecule drugs, large molecule drugs, conjugated nanoparticles, and chemical exposures. Alternative approaches to determine PK have been explored, including physiologically based PK modeling, *in vitro* methods, and traditional animal models. These alternative approaches along with convenience sampling of tissue or fluid biospecimens can address challenges in studying maternal-fetal pharmacology. In this narrative review, we 1) present an overview of the current understanding of maternal-fetal drug exposure; 2) discuss biospecimen-guided sampling design and methods for measuring fetal drug concentrations throughout gestation; and 3) propose methods for advancing pharmacology research in the maternal-fetal population.

## Introduction

Prescription medication use during pregnancy is widespread. At least 70% of individuals take at least one prescription medication during pregnancy (Lupattelli et al., 2014; Haas et al., 2018; Centers for Disease Control and Prevention, 2022). These medications may be prescribed to treat an individual’s chronic conditions (e.g., depression, epilepsy, hypertension, thyroid disorders), acute illnesses (e.g., infections), and pregnancy-related illnesses (e.g., pre-eclampsia or gestational diabetes) (Wesley et al., 2021). Many of these drugs will cross the placenta and expose the fetus. The extent and impact of fetal exposure is unknown for most drugs.

In order to optimize drug dosing in pregnant individuals and prevent harm to the fetus, it is critical to understand physiologic changes during pregnancy that determine fetal drug exposure. However, determining fetal drug exposure is challenging. *In utero* sampling procedures to directly measure fetal drug concentrations are invasive and place both mother and baby at increased risk for adverse events. Preclinical *in vitro* and animal models are not always translatable to humans. Opportunistic samples obtained during prescribed clinical care leverages standard of care procedures (e.g., collecting amniotic fluid at the time of routine amniocentesis) and collect non-invasive surrogate samples related to fetal exposure (e.g., fetal hair or meconium) as an alternative approach to assessing fetal drug transfer.

When formulating this manuscript, we essentially wanted to answer the question, “How do we obtain drug levels from pregnant individuals for clinical studies or trials to measure fetal drug exposure?” To help answer this, we provide narrative for the current understanding of maternal-fetal drug transfer, evaluate the pros and cons of different opportunistic sampling approaches, and investigate potential alternative methods to better characterize fetal pharmacology.

## Current understanding of maternal-fetal drug transfer

### Maternal anatomic and physiological changes during pregnancy

Human gestation length is about 280 days and is divided into three trimesters. The first trimester is usually dated from the start of the mother’s last menstrual period, which is 2 weeks before the estimated date of conception, and continues through week 12. This is often designated as the embryonic period. The second trimester comprises the most prolonged period and is defined as weeks 13–28. The third trimester begins at week 29 and continues until delivery, typically at week 40 for a full term delivery (Andersen et al., 2018). Each trimester is marked by maternal changes in anatomy and physiology, such as renal function. For example, the glomerular filtration rate and renal plasma flow increase up to 50% and 80%, respectively, during pregnancy (Cheung and Lafayette, 2013). As another example, increases in estradiol and progesterone are initiated at the beginning of pregnancy and are regulated by the placenta starting at week 10 (Weissgerber and Wolfe, 2006; Kumar and Magon, 2012). Pregnancy related changes in these hormones can, both directly and indirectly, affect the pharmacokinetics (PK) of drugs through competition for binding to plasma proteins, changes in the activity of metabolic enzymes (Table 1), and other anatomical and physiological changes such as changes in gastrointestinal motility. (Dickinson et al., 1989; Gerdin et al., 1990; Prevost et al., 1992; Tomson et al., 1994; Hakkola et al., 1996; Collier et al., 2002; McGready et al., 2003; Nishimura et al., 2003; De Haan et al., 2004; Dempsey et al., 2004; Franco et al., 2008; Hebert et al., 2008; Ke et al., 2014; Fa et al., 2018; Goh et al., 2021). These types of changes can affect drug absorption, distribution, metabolism, and excretion (ADME) as highlighted in Table 2 (Ke et al., 2014; Feghali et al., 2015; Kazma et al., 2020).

**TABLE 1:** Summary of drug metabolizing enzyme activity by gestational age.

Metabolizing enzyme	Change in activity during gestation by trimester			Expressed in placenta	References
	First^a^	Second^a^	Third^a^		
CYP1A1	-	-	-	yes	Collier et al. (2002), Ke et al. (2014), Fa et al. (2018), Goh et al. (2021)
CYP1A2	+	-	-	no	Hakkola et al. (1996), Nishimura et al. (2003), Goh et al. (2021)
CYP2C9	+	+	+	yes	Dickinson et al. (1989), Tomson et al. (1994)
CYP2C19		-	-	yes	McGready et al. (2003), Ke et al. (2014)
CYP2A6		+	+	no	Dempsey et al. (2004)
CYP2B6		+	+	no	Hakkola et al. (1996), Ke et al. (2014)
CYP3A4	+	+	+	no	Prevost et al. (1992), Hebert et al. (2008)
CYP2D6	+	+	+	yes	Hakkola et al. (1996)
UGT1A4	+	+	+	yes	Collier et al. (2002), De Haan et al. (2004), Franco et al. (2008)
UGT2B7			+	yes	Gerdin et al. (1990), Collier et al. (2002)

^a^ Blank spaces indicate no information found for metabolizing enzyme expression in indicated gestational trimester; - indicates a decrease; + indicates an increase.

**TABLE 2:** Selected maternal organ system changes that affect pharmacokinetics during pregnancy.

Specific organ system	Change during pregnancy	PK effect	References
Renal plasma flow	Increase (up to 80%)	Increase CL	Feghali et al. (2015), Kazma et al. (2020)
Glomerular filtration rate	Increase (up to 50%)	Increase CL	Kazma et al. (2020)
Gastrointestinal tract motility	Decrease (not reported)	Delay K_a_	Feghali et al. (2015), Kazma et al. (2020)
Cardiac output	Increase (20%–45%)	Increase K_a_ and V_d_	Feghali et al. (2015)
Creatinine clearance	Increase (26%–28%)	Increase CL	Ke et al. (2014)
Uterine blood flow	Increase (923%–2,721%)	Increase K_a_	Ke et al. (2014)
Total fat mass	Increase (6%–23%)	Increase V_d_	Ke et al. (2014)

*CL*, clearance, *K*_*a*_, absorption; *PK*, pharmacokinetic, *V*_*d*_, volume of distribution.

### Fetal drug exposure

**FIGURE 1:**
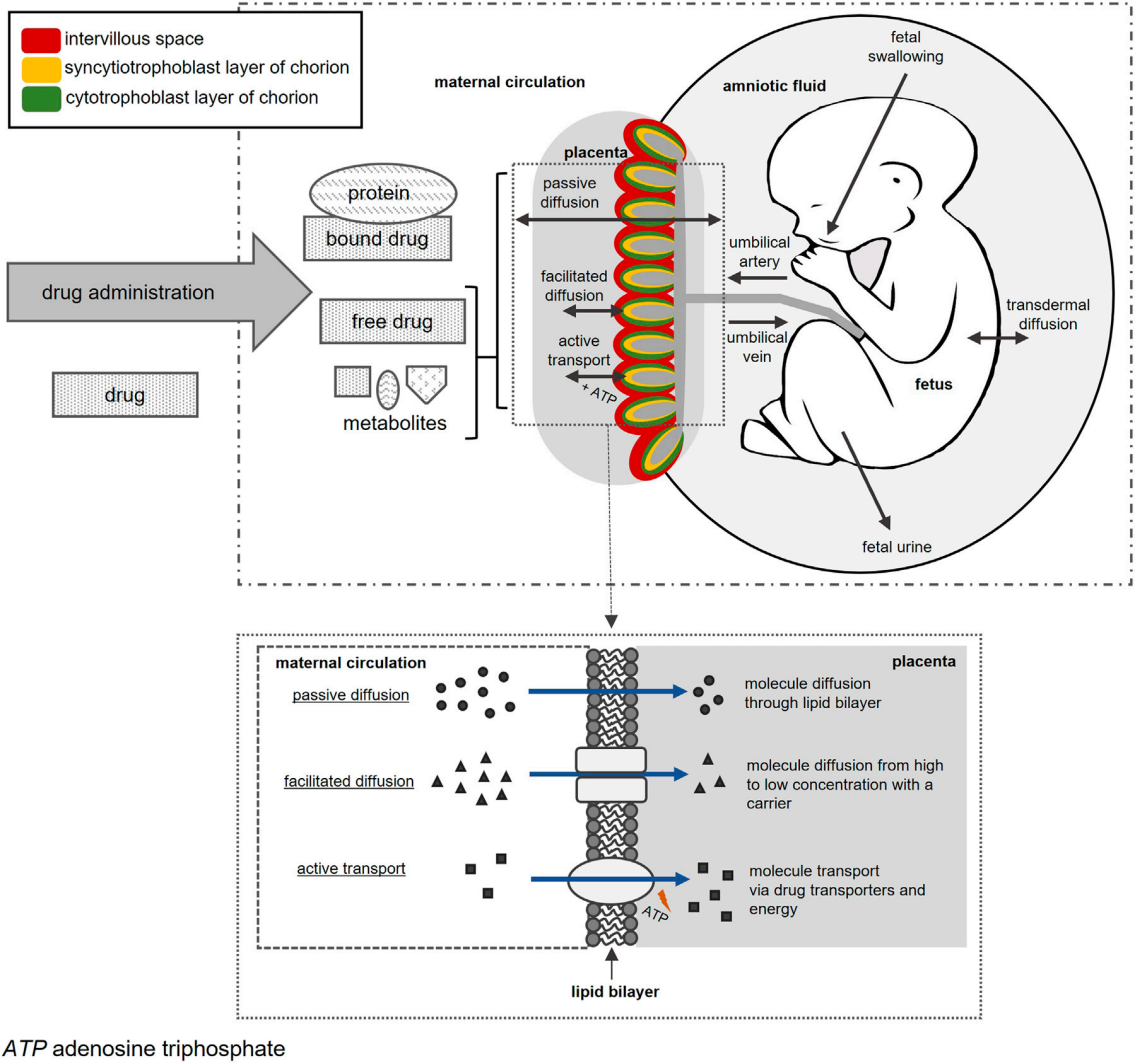
*In utero* fetal drug transfer following maternal drug administration.

The placenta performs vital functions for the developing fetus and has several structural components. The basic structural unit of this disk-shaped organ is the chorionic villi that project into the intervillous space (Griffiths and Campbell, 2015). Chorionic villi are surrounded by the chorion which consists of the outer syncytiotrophoblast and inner cytotrophoblast layers (Griffiths and Campbell, 2015). Placental structural components and activity are vital for normal embryonic development to ensure sufficient oxygen, nutrient, and waste exchange between mother and fetus (Grigsby, 2016). Maternal-fetal drug exposure and PK are largely moderated by the placenta. Drugs in maternal blood can reach fetal blood by passing through the placental intervillous space, syncytiotrophoblast layer, and fetal connective tissue to reach the endothelium of fetal capillaries and enter the fetal circulation (Figure 1) (Griffiths and Campbell, 2015). Drugs in fetal circulation can also re-enter maternal blood in small amounts (Syme et al., 2004; Griffiths and Campbell, 2015).

Placental transfer of drugs can occur *via* passive diffusion, facilitated diffusion, or active transport (Griffiths and Campbell, 2015; Pemathilaka et al., 2019). Minute transfer may occur *via* pinocytosis and phagocytosis, but these mechanisms are too slow to play a significant effect on fetal drug concentrations (Syme et al., 2004). Passive diffusion of drugs occurs for neutral, lipophilic, and unbound drugs with a molecular weight less than 500 Daltons (Pavek et al., 2009a; Feghali et al., 2015). Facilitated diffusion occurs when drugs are structurally related to endogenous compounds such as glucocorticoids (Griffiths and Campbell, 2015; Pemathilaka et al., 2019). Drug transporters, such as multidrug resistance proteins (MRPs), P-glycoprotein (P-gp), and breast cancer resistance protein (BCRP), require energy, usually in the form of adenosine triphosphate, to actively transfer drugs (Myllynen et al., 2009; Iqbal et al., 2012; Griffiths and Campbell, 2015; Pemathilaka et al., 2019). Drug transporters present in the placenta allow drug transfer from mother to fetus and *vice versa* (Griffiths and Campbell, 2015).

Placental transfer of drugs can be further complicated as the placenta contains a broad range of enzymatic activity (Prouillac and Lecoeur, 2010). Several cytochrome P450 (CYP) drug metabolizing enzymes have been isolated from the placenta and include CYP1, CYP2, and CYP3 (Myllynen et al., 2009; Prouillac and Lecoeur, 2010). These enzymes, along with active drug transporters, alter fetal exposure to varying amounts of parent drug, metabolites, and byproducts (Dallmann et al., 2019a).

The importance of placental effects is exemplified by a study that investigated illicit drug exposure in monozygotic and dizygotic twins (Boskovic et al., 2001). Similar concentrations of cocaine and cannabinoids were found for monozygotic twins who share the same placenta. More significant differences in drug concentrations were observed in dizygotic twins with separate placentas. Notably, one dizygotic twin tested positive for drugs while the other twin did not. This study demonstrates the variation in drug transfer across the placenta that can alter fetal concentrations.

Once a drug reaches the fetus, fetal ADME can impact fetal drug exposure. Fetal ADME differs substantially from maternal ADME and even infant ADME (Feghali et al., 2015; Allegaert and Van Calsteren, 2016). For example, expression levels of fetal CYP enzymes mature over the course of pregnancy and, in general, are much lower than infant and maternal expression levels (Lacroix et al., 1997; Hines, 2008). In addition, drugs and metabolites can become trapped in fetal tissues *via* two processes: 1) reabsorption from amniotic fluid and 2) ionization. First, drugs that are renally excreted by the fetus can recirculate through the amniotic fluid and be reabsorbed through fetal swallowing (Pritchard, 1966; Blackburn and Loper, 1992; Pavek et al., 2009b; Abduljalil et al., 2019). The fetal swallow reflex begins as soon as week 10 of gestation (De Vries et al., 1985). Second, he pH of fetal blood is slightly more acidic than maternal blood leading to ionization of weak bases. When ionized, these weak bases usually do not pass from the fetus back to the mother *via* the placenta (Pavek et al., 2009b). These fetal-specific aspects confound generalizations and complicate measurement of fetal drug exposure.

## Fetal drug detection from biological fluid and tissue specimens

**FIGURE 2:**
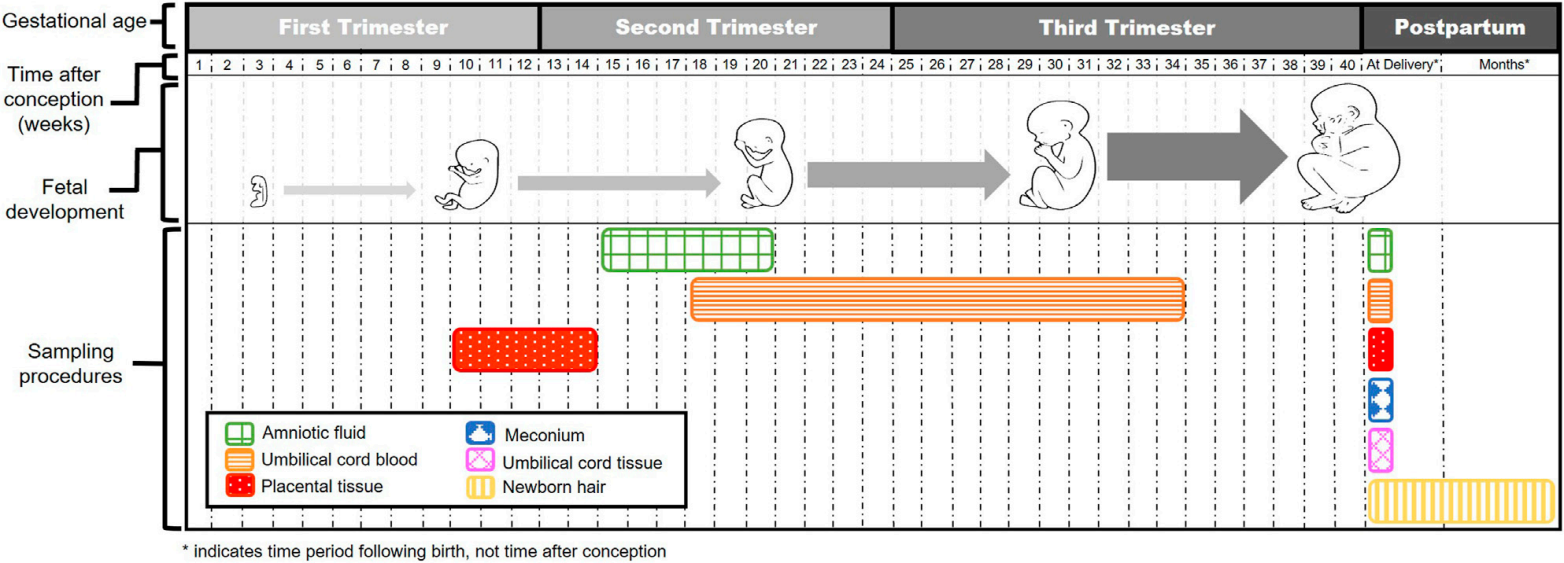
Illustration of surrogate sampling times throughout the stages of gestation.

Methods for measuring fetal drug concentrations are invasive in nature and pose risks to both mother and fetus. To minimize risks, the collection of opportunistic surrogate samples during standard of care procedures increases feasibility for measuring fetal drug exposure. Several of these surrogate options are illustrated in Figure 2.

### Amniotic fluid

#### Background and sampling

Amniotic fluid provides protection and temperature regulation during fetal development, and its composition changes as pregnancy progresses (Beall et al., 2007). During early embryogenesis before fetal kidneys start to function, amniotic fluid is predominately thought to derive from maternal plasma (Beall et al., 2007; Orczyk-Pawilowicz et al., 2016). Its composition shifts following the first trimester with increased creatinine, urea, and uric acid concentrations, most likely a consequence of fetal swallowing and renal excretion (Brace and Wolf, 1989; Bloomfield et al., 2017). *In utero* sampling of amniotic fluid, known as amniocentesis, is performed for specific diagnostic testing. Amniocentesis is typically conducted after weeks 15–16 of gestation when the amnion and chorion have fused (Jindal and Chaudhary, 2020). Other options for obtaining amniotic fluid would be in cases of miscarriage, planned termination of pregnancy, or at delivery.

#### Maternal-fetal drug transfer

Drug concentrations have been evaluated in amniotic fluid from early and mid-gestation as well as at delivery (Table 3) (Bernard et al., 1977a; Bernard et al., 1977b; Szeto et al., 1978; Mandelbrot et al., 2001; Chappuy et al., 2004a; Chappuy et al., 2004b; Fokina et al., 2016; Paulzen et al., 2017a; Paulzen et al., 2018; Paulzen et al., 2020). While amniocentesis is not typically carried out prior to week 15 of gestation, drug concentrations in amniotic fluid have been reported during the first trimester from older practices. Dependent on gestational age and drug evaluation, conflicting results are reported between drug concentrations in amniotic fluid versus fetal tissue, fetal plasma, and maternal blood. For example, diclofenac and amikacin concentrations measured in amniotic fluid were lower than concentrations measured in fetal tissue samples. In contrast, ritodrine and quetiapine concentrations measured in amniotic fluid and umbilical cord blood were similar at delivery (Bernard et al., 1977a; van Lierde and Thomas, 1982; Siu et al., 2000; Paulzen et al., 2018). These discrepancies highlight crucial factors when considering amniotic fluid as a biospecimen, including drug permeability to fetal skin, amniotic fluid composition, and effects of fetal and maternal hepatic metabolism throughout pregnancy (Ward and Varner, 2019).

**TABLE 3:** Description of studies that reported drug concentrations in surrogate specimens by gestational age and at delivery.

Surrogate specimen	Gestational age by trimester				References
	Frist^a^	Second^a^	Third^a^	At delivery^a^	
Amniotic fluid	x	x		x	Bernard et al. (1977a), Bernard et al. (1977b), Szeto et al. (1978), Pons et al. (1991), Siu et al. (2000), Mandelbrot et al. (2001), Chappuy et al. (2004a), Chappuy et al. (2004b), Fokina et al. (2016), Paulzen et al. (2017a), Paulzen et al. (2018), Paulzen et al. (2020)
Umbilical cord blood	x	x		x	Kauffman et al. (1975), Bernard et al. (1977a), Bernard et al. (1977b), Pons et al. (1991), Mandelbrot et al. (2001), Hendrick et al. (2003), Paulzen et al. (2017a), Paulzen et al. (2017b), Veit et al. (2017), Paulzen et al. (2018), Paulzen et al. (2020)
Placental tissue	x	x		x	Bernard et al. (1977a), Bernard et al. (1977b), de Barros Duarte et al. (2009), Duarte et al. (2011)
Meconium				x	Ostrea et al. (1989), Maynard et al. (1991), Ostrea et al. (2001), Bar-Oz et al. (2003), Eyler et al. (2005), Montgomery et al. (2006), Gray and Huestis (2007), Montgomery et al. (2008), Concheiro et al. (2010), Marin et al. (2014), Colby (2017)
Umbilical cord tissue				x	Montgomery et al. (2006), Montgomery et al. (2008), Concheiro et al. (2010), Concheiro et al. (2013), Marin et al. (2014), Colby (2017)
Newborn hair				x	Eliopoulos et al. (1996), Klein and Koren (1999), Boskovic et al. (2001), Ostrea et al. (2001), Bar-Oz et al. (2003), Gray and Huestis (2007)

^a^ Blank spaces indicate no studies found for surrogate specimens; x indicates reported surrogate specimen analysis was reported for the trimester.

#### Limitations

Amniocentesis is an invasive test and carries certain risks to the mother and fetus. Risks of mid-trimester amniocentesis include rupturing the amniotic sac, miscarriage, needle injury to the fetus, Rh sensitization, and infection (Pruthi, 2020). Amniocentesis before week 15 of gestation is associated with a higher rate of miscarriages than mid-term amniocentesis and is rarely performed unless the benefits outweigh the risks (Wilson, 1995; Steinfort et al., 2021). With advancing gestation, additional risks include preterm birth, chorioamnionitis, and stillbirth (Daum et al., 2019). Therefore, amniocentesis would only be viable option in cases where an amniocentesis was performed for clinical indications. In these cases, the fetus often has anomalies or suspected genetic abnormalities, which may influence drug metabolism. Because amniocentesis is typically carried out mid-gestation, sampling opportunities may be limited during early and late pregnancy.

### Umbilical cord blood

#### Background and sampling

The umbilical vein provides blood from mother to fetus with flow established within the umbilical cord by the end of week 5 of gestation (Spurway et al., 2012). Umbilical cord blood has a unique composition as it contains blood cells with varying stem cell markers, and differs from both newborn and maternal peripheral blood (Pranke et al., 2001). Composition is also influenced by fetal sex, gestational age, and mode of delivery (Glasser et al., 2015). Fetal gender appears to influence red blood cell values and white blood cells are reported to increase with gestational age and vaginal births (Glasser et al., 2015). Cord blood can be collected *in utero* (cordocentesis-usually of the fetal vein), typically between week 18–34 of gestation, and at the time of delivery (Jindal and Chaudhary, 2020). Other options for obtaining cord blood would be in cases of miscarriage, planned termination of pregnancy, or at delivery.

#### Maternal-fetal drug transfer

Umbilical cord blood measurements are predominantly reported mid-to late-gestation or at delivery (Kauffman et al., 1975; Pons et al., 1991; Mandelbrot et al., 2001; Hendrick et al., 2003; Paulzen et al., 2017a; Paulzen et al., 2017b; Paulzen et al., 2018; Paulzen et al., 2020). Most studies assumed cord blood was informative of fetal exposure. This assumption is supported by one study that measured similar gentamicin concentrations in fetal and cord serum following elective second trimester abortion (Kauffman et al., 1975). However, most of the reported studies only compared cord blood measurements with maternal serum. Because of differences in PK between the mother and the fetus, single pairs of samples from the mother and the umbilical cord blood can show ratios that vary widely depending on the interval after drug administration (Ward, 1995). Data evaluating the relationship between drug concentrations in cord blood and other fetal samples may provide further insight into fetal exposure.

#### Limitations

Cordocentesis is an invasive test with risks to the pregnancy. Procedure-related risks include bleeding from the puncture site (most common), fetal distress, pregnancy loss, and rarely vertical transmission of maternal infection (Society for Maternal-Fetal et al., 2013). Therefore, cordocentesis would only be a viable option when performed for clinical indications. In these cases, the fetus may have anemia, which may influence the activity of enzymes involved in drug metabolism. Like amniocentesis, cordocentesis is typically carried out mid-gestation, but can extend to late pregnancy. Technical aspects of cordocentesis limit its use during early pregnancy.

### Placental tissue

#### Background and sampling

Placental chorionic villi serve as the functional and structural unit of the human placenta and are involved in the exchange of gas and nutrients between mother and fetus (Gude et al., 2004). During fetal development, chorionic villi grow and form branches as pregnancy progresses with high variability in vascularization, the degree of branching, and budding (Gude et al., 2004; Hannibal et al., 2018). Chorionic villus sampling (CVS) is conventionally conducted between weeks 10 and 14 during the first trimester (Jindal and Chaudhary, 2020). Other options for obtaining a chorionic villus biospecimen would be in cases of miscarriage, planned termination of pregnancy, or at delivery.

#### Maternal-fetal drug transfer

Studies to evaluate drug concentrations from the placental tissue by CVS have not been explored extensively. Some studies have evaluated concentrations of bupivacaine enantiomers, lidocaine, and fentanyl from the placental intervillous space following term deliveries (de Barros Duarte et al., 2009; Duarte et al., 2011). While these studies reported relatively high drug and drug metabolite concentrations, the translation of this work to chorionic villi samples rather than placental intervillous space is uncertain. In addition, CVS is typically conducted in early gestation, and the cited studies were carried out in late gestation following term deliveries. Measuring drug concentration in CVS biospecimens should be explored for estimating fetal drug exposure in the first trimester of pregnancy using convenience samples obtained as part of clinically indicated sampling.

#### Limitations

CVS is an invasive test with risks to pregnancy. Risks of CVS include infection, membrane rupture, and fetal loss (Jindal and Chaudhary, 2020). Therefore, collection of chorionic villi biospecimens is only an option in cases where a CVS is performed for clinical indications. This restricts *in utero* CVS biospecimen collection to early pregnancy. Overall, our understanding of drug concentrations measured from chorionic villi are quite limited.

### Meconium

#### Background and sampling

Meconium is the initial substance present in the intestines of a developing fetus and constitutes the first stools of a newborn (Skelly et al., 2020). Meconium accumulates during the second trimester (weeks 13–16) when fetal swallowing begins (Skelly et al., 2020). Drug concentrations detected in meconium represent cumulative exposure from the second trimester through birth. Collection of meconium can typically be conducted within the first 24 to 48 h following birth dependent on the timing of the first newborn stool (Skelly et al., 2020).

#### Maternal-fetal drug transfer

Meconium is frequently used for detecting fetal drug exposure concentrations in newborns for suspected maternal illicit drug use. It has been studied extensively (Ostrea et al., 1989; Maynard et al., 1991; Ostrea et al., 2001; Bar-Oz et al., 2003; Eyler et al., 2005; Montgomery et al., 2006; Gray and Huestis, 2007; Montgomery et al., 2008; Concheiro et al., 2010; Marin et al., 2014; Colby, 2017). Although used extensively to detect illicit perinatal drug use, the convenience of this sampling supports the use of this biospecimen to determine *in utero* fetal drug transfer of non-illicit drugs. Meconium has been recognized as a sensitive biospecimen to detect *in-utero* drug exposure (Ostrea et al., 2001; Bar-Oz et al., 2003; Eyler et al., 2005; Gray and Huestis, 2007).

#### Limitations

Sampling of meconium can be limited if meconium is passed early *in utero* before birth (Farst et al., 2011). Meconium is also frequently contaminated with urine from diaper collection, complicating drug concentration interpretation (Gray and Huestis, 2007). While meconium sampling offers a wide window of drug detection, it is impossible to distinguish a single concentration time-point of drug exposure (Gareri et al., 2006). Drug concentrations measured in meconium represent the accumulation of drug exposure *in utero* over many weeks to months. Drug use just prior to delivery may not have had time to distribute and thus may relay inaccurate results (Farst et al., 2011). Furthermore, it is not clear when during pregnancy drugs first appear in meconium, or how the meconium concentration compares to the extent of maternal drug use.

### Umbilical cord tissue

#### Background and sampling

The umbilical cord provides a pathway for blood transport from the placenta to the fetus (Spurway et al., 2012). Development of the umbilical cord begins between weeks 4 and 8 of pregnancy with the amnion enveloping tissue from the body stalk (Schöni-Affolter et al., 2007; Spurway et al., 2012). As an option for monitoring *in utero* fetal drug exposure, cord tissue can be collected following birth. Collection of cord tissue can be conducted relatively quickly as it does not require an invasive procedure, utilizes an otherwise discarded specimen, and may reflect a relatively long window of drug detection (Price et al., 2020).

#### Maternal-fetal drug transfer

Several studies have compared samples from the umbilical cord tissue versus meconium to assess fetal concentrations following prescribed medication intake and illicit drug use (Montgomery et al., 2006; Montgomery et al., 2008; Concheiro et al., 2010; Concheiro et al., 2013; Marin et al., 2014; Colby, 2017). Among these studies, investigators have suggested similar sensitivity and specificity between meconium and cord tissue, yet cord tissue may offer some advantages. For example, meconium collection varies based on newborn passage while cord tissue can be sent for testing immediately following delivery (Montgomery et al., 2006). Cord tissue has been utilized in standard clinical practice for estimating fetal drug exposure, which supports its use as a suitable biospecimen.

#### Limitations

Umbilical cord tissue sampling can only be performed following birth or termination of pregnancy. This results in a significant limitation in sampling, with no ability to use cord tissue when conducting fetal drug exposure analysis before birth. An important consideration for use of cord tissue is the possibility for drug metabolites to passively diffuse from cord plasma to cord tissue *in utero* and confound measured drug concentrations (Concheiro et al., 2010). Several studies reported possible “false negatives” from cord tissue because drug metabolites were found rather than the parent compound. Therefore, variations in maternal and fetal kinetic patterns suggest cord tissue drug concentrations may not accurately reflect the extent of maternal to fetal drug transfer (Ward, 1995).

### Neonatal hair

#### Background and sampling

Fetal hair aids in *utero* skin protection and temperature regulation. Hairs project from all skin surface areas and the hair shaft becomes fully formed by the beginning of the third trimester (Holbrook and Odland, 1978). The foremost advantage of fetal hair as a biospecimen is its collection at any point during the first 3 months of life. After 3 months, neonatal hair is replaced with infant hair (Gray and Huestis, 2007).

#### Maternal-fetal drug transfer

Neonatal hair testing has identified fetal drug exposure from specific drugs of abuse (Eliopoulos et al., 1996; Klein and Koren, 1999; Boskovic et al., 2001; Ostrea et al., 2001; Bar-Oz et al., 2003; Gray and Huestis, 2007). A high correlation was reported for drug concentrations in paired maternal and neonatal hair specimens (Klein and Koren, 1999). These concentrations would be reflective of drug exposure relatively late in pregnancy as fetal hair grows during the third trimester.

#### Limitations

Similar to meconium and cord tissue, neonatal hair can only be collected following birth. Sampling may be limited in newborns born with limited hair or baldness (Gray and Huestis, 2007). In some cases, mothers are unwilling to consent to fetal hair collection for cosmetic or cultural reasons (Gray and Huestis, 2007). Drug concentrations measured in neonatal hair represent the accumulation of drug exposure *in utero* relatively late in pregnancy. It is not possible to distinguish a single concentration time-point of drug exposure. Furthermore, differing amounts of melanin in neonatal hair may confound measured drug concentrations. Higher amounts of melanin present in dark colored hair can bind more drug than lighter colored hair (Slawson et al., 1998).

## Alternative approaches to estimate maternal-fetal drug transfer

While *in utero* PK studies are ideal, decreases in prenatal testing limit access to biospecimens collected before birth. The difficulties associated with biological fluid and tissue sampling during pregnancy have motivated the development of alternative methods to study fetal drug exposure.

### Physiologically based pharmacokinetic (PBPK) modeling

#### Background

PBPK models are mathematical tools that integrate drug-specific information (e.g., metabolism, protein binding) and system-specific information (e.g., organ size, blood flow) to predict the effect of physiological conditions (e.g., pregnancy) on drug exposure (Edginton et al., 2006; Zhao et al., 2011; Dallmann et al., 2019a; Dallmann et al., 2019b; Silva et al., 2022). To model drug exposure in pregnant individuals, pregnancy-related virtual organs can be linked to the PBPK model. Model parameters (e.g., increased GFR) can then be modified to reflect pregnancy physiology (Dallmann et al., 2018). One advantage of PBPK models includes the ability to use published or opportunistic PK study data to predict fetal drug exposure. This combined approach allows for the simulation of clinical trials, improved trial design, and reduced number of pregnant individuals needed for PK dosing studies.

#### Maternal-fetal drug transfer

Pregnancy PBPK models have demonstrated excellent capabilities in the last few decades as predictive tools for maternal and fetal populations. These models build on existing information and data to describe maternal-fetal drug transfer throughout pregnancy. There is an increasing focus on methodologies for including placental transfer physiology to describe fetal exposure (De Sousa Mendes et al., 2017; Zhang et al., 2017; Zhang et al., 2018; George et al., 2020; Liu et al., 2020; Mian et al., 2020; Gingrich et al., 2021; Abduljalil et al., 2022a; Bukkems et al., 2022; Peng et al., 2022). Methodologies capitalize on available *in vitro*, *in vivo*, and *ex vivo* studies in animals and humans to inform models for fetal exposure. These combined advancements have allowed for the consolidation of physiological changes into reference databases for pregnancy models (Dallmann et al., 2017; Dallmann et al., 2019a; Abduljalil et al., 2022b). PBPK models and databases provide a quantitative framework for placental transfer and examining fetal exposure throughout pregnancy. This framework has the flexibility to incorporate changes in drug-specific and physiology-specific components to advance our understanding of maternal PK and fetal drug exposure.

#### Limitations

PBPK model validation still requires biologic sampling. While smaller sample sizes are required for PBPK modeling, pronounced physiological changes necessitate dynamic assumptions for model building. Additional data are needed throughout gestation to improve model accuracy, build inter-individual and intra-individual variability, and validate the PBPK models (Center for Drug Evaluation and Research, 2019).

### Placenta-on-a-chip

#### Background

The placenta is responsible for regulating drug transfer to the fetus during pregnancy. To explore this, a “placenta-on-a-chip” system that mimics the structure and function of the human placenta has been assessed. This microdevice concept typically includes the static culture of trophoblast monolayers in Transwell inserts to mimic the placental passage of compounds (Poulsen et al., 2009). Some advanced models include human trophoblast cells and villous endothelial cells cultured in apposition on a semipermeable membrane under flow conditions (Blundell et al., 2018). This *in vitro* device offers the opportunity to carry out non-invasive experiments that do not interfere with the care of the mother or fetus.

#### Maternal-fetal drug transfer

An advanced placenta-on-a-chip model has been developed to study transporter-mediated drug efflux. The placental barrier’s multilayered architecture and hemodynamic environment were mimicked with a single device *in vitro* (Blundell et al., 2018). Examination of the model assessing glyburide transfer was consistent with some *in vivo* studies (Elliott et al., 1991; Langer et al., 2000). This model for drug transfer is appealing as it gains the capacity to precisely control and manipulate critical parameters of placental drug transport. Placenta-on-a-chip models have explored the transfer of other compounds, including caffeine and nanoparticles (Nadanaciva et al., 2011; Pemathilaka et al., 2019).

#### Limitations

These studies offer reasonable contributions to assessing the maternal-fetal transfer of different compounds using *in vitro* strategies; however, additional research is needed to confirm these models. Future development requires the incorporation of changes in drug transporters and metabolizing enzymes throughout gestation.

## Discussion

A better understanding of maternal-fetal pharmacology is critical for both the mother and fetus. Changes in anatomy and physiology during pregnancy can result in supra- or subtherapeutic dosing. In current practice, dosage adjustments for medications during pregnancy are rare due to limitations in literature and dosing guidance. Dosing adjustments may be necessary for drugs that put the fetus at increased risk. In particular, additional data are needed for drugs or medications that concentrate in the fetal compartment. Further investigation of fetal drug PK in pregnancy is a priority area with implications for both mother and fetus.

Improved methods and protocols are needed to collect concentration data throughout gestation. Convenience sampling is a method that would allow sample collection during already indicated *in utero* procedures. By utilizing multiple procedures, concentration time-point measurements can be collected during each trimester. For example, CVS is typically conducted in the first trimester, while amniocentesis is carried out during the second trimester and cordocentesis is available in the early third trimester. Further collection of these biospecimens in addition to cord tissue, meconium, and neonatal hair at or after delivery can provide additional PK data. Incorporation of PK data with alternative approaches can inform fetal drug exposure.

Given the inherent limitations of invasive sampling, alternative approaches are necessary to supplement our understanding of fetal drug disposition. Examples of alternative approaches include traditional animal models as well as *in silico* and *in vitro* methods. Historically, animal models have been utilized to study the passage of drugs from mother to fetus, yet these results are not always transferrable to humans (Bracken, 2009). Animal placental anatomy, gestation lengths, and translatability to the clinical setting should be considered when using this approach (Grigsby, 2016). PBPK modeling used to describe medications administered during pregnancy is becoming more popular, but data to validate this approach is essential. In attempt to provide pregnancy exposure data to the public, the FDA organizes registries that collect information on exposure to medical products during pregnancy (Food and Drug Administration, 2023). However, limited concentration data for validation of fetal exposure is available through these post-marketing registries. It is therefore necessary to supplement this data with well-designed, opportunistic trials as well as share study results from academic and government institutions. Placenta-on-a-chip and other experimental *in vitro* approaches (Myllynen and Vahakangas, 2013) have the potential to provide important information; however, these techniques currently lack integration of changes that occur throughout pregnancy. Excellent examples of combining multiple approaches to estimate human fetal drug exposure have recently been published (Balhara et al., 2022; Roelofsen et al., 2022). Alternative approaches can provide insight into fetal drug exposure during human pregnancy and inform dosing in clinical trials that include pregnant individuals.

The importance of including pregnant individuals in drug therapy studies cannot be overstated. The U.S. Food and Drug Administration (FDA) has recently focused attention on the importance of including pregnant individuals in clinical trials (Vasisht et al., 2021), and drawn attention to their extensive Final Rule on drug labeling for use during pregnancy (Food and Drug Administration, 2014). In addition, the FDA recommends that clinical research including pregnant individuals meet all ten conditions specified in the U.S. Department of Health and Human Services regulations (Food and Drug Administration, 2018). These regulations acknowledge the variations in local regulations involving pregnant minors in pregnancy-related research and outline how to consider risks to both the mother and fetus. Regulations for considering the fetal effects of new drugs are extremely limited, as pediatric regulations (21 CFR subpart D) do not apply to the fetus (Green et al., 2021). Only U.S. Health and Human Service regulations (45 CFR Part 46) apply to the fetus (Green et al., 2021). Rules and regulations from the FDA, European Medicines Agency, and other agencies outline ethical considerations associated with conducting clinical trials involving pregnant individuals (European Medicines Agency, 2005; International Council for Harmonisation of Technical Requirements for Pharmaceuticals for Human Use (ICH), 2016; Food and Drug Administration, 2022).

Ethical considerations in fetal medicine are complex, involving the interests of the mother, the father, and the fetus. Medications administered to the mother during pregnancy cross the placenta to reach the fetus in varying amounts. Fetal exposure may be below or above the NOAEL (no observable adverse effect level), which is the threshold for an adverse fetal effect. Prospective studies to determine NOAEL without therapy intended to benefit the mother, fetus, or both is unethical. The extent of maternal drug disposition and the amount of maternal-fetal drug transfer varies for specific pathways throughout pregnancy. By utilizing available data, convenience biospecimen sampling, and alternative approaches, we can optimize clinical care and minimize risk to the mother and fetus during pregnancy.

## Conclusion

Ethical considerations are unavoidable when considering pregnant individuals in clinical trials and research studies. Notably, anatomical and physiological changes throughout pregnancy can impact risk associated with medication or illicit drug use. Here, we present different sampling options from various biospecimens *in utero* and following birth to aid in quantifying maternal-fetal drug transfer. Biospecimen samples may opportunistically be collected during a procedure for a prenatal standard of care medical decision. Non-invasive approaches, including animal models, PBPK modeling, and *in vitro* methods, provide a gateway for scientists to explore fetal drug transfer without putting the mother or fetus at risk. These and other innovative methods are necessary to advance the field of maternal-fetal pharmacology.

Nonetheless, future exploration is necessary when investigating medications in pregnant populations.
